# *WHITE STRIPE LEAF4* Encodes a Novel P-Type PPR Protein Required for Chloroplast Biogenesis during Early Leaf Development

**DOI:** 10.3389/fpls.2017.01116

**Published:** 2017-06-26

**Authors:** Ying Wang, Yulong Ren, Kunneng Zhou, Linglong Liu, Jiulin Wang, Yang Xu, Huan Zhang, Long Zhang, Zhiming Feng, Liwei Wang, Weiwei Ma, Yunlong Wang, Xiuping Guo, Xin Zhang, Cailin Lei, Zhijun Cheng, Jianmin Wan

**Affiliations:** ^1^National Key Facility for Crop Gene Resources and Genetic Improvement, Institute of Crop Science, Chinese Academy of Agricultural SciencesBeijing, China; ^2^National Key Laboratory for Crop Genetics and Germplasm Enhancement, Nanjing Agricultural UniversityNanjing, China

**Keywords:** chloroplast, *Oryza sativa* L., pentatricopeptide repeat, RNA splicing, WSL4

## Abstract

Pentatricopeptide repeat (PPR) proteins comprise a large family in higher plants and perform diverse functions in organellar RNA metabolism. Despite the rice genome encodes 477 PRR proteins, the regulatory effects of PRR proteins on chloroplast development remains unknown. In this study, we report the functional characterization of the rice *white stripe leaf4* (*wsl4*) mutant. The *wsl4* mutant develops white-striped leaves during early leaf development, characterized by decreased chlorophyll content and malformed chloroplasts. Positional cloning of the *WSL4* gene, together with complementation and RNA-interference tests, reveal that it encodes a novel P-family PPR protein with 12 PPR motifs, and is localized to chloroplast nucleoids. Quantitative RT-PCR analyses demonstrate that *WSL4* is a low temperature response gene abundantly expressed in young leaves. Further expression analyses show that many nuclear- and plastid-encoded genes in the *wsl4* mutant are significantly affected at the RNA and protein levels. Notably, the *wsl4* mutant causes defects in the splicing of *atpF, ndhA, rpl2*, and *rps12*. Our findings identify WSL4 as a novel P-family PPR protein essential for chloroplast RNA group II intron splicing during early leaf development in rice.

## Introduction

Chloroplasts, thought to have originated from cyanobacteria through endosymbiosis, are the exclusive organelles for photosynthesis in plants and algae, and also have important roles in synthesis and storage of many key metabolites, such as lipids, terpenoids, and amino acids (Mullet, 1993; Moreira et al., 2000; Sugimoto et al., 2004). Extensive studies have uncovered the basic photosynthetic and metabolic processes of chloroplast development (Kangasjärvi et al., 2014; Kaur, 2014; de Souza et al., 2016).

Chloroplast development from proplastids is subdivided into three stages; that are coordinately regulated by both plastid- and nuclear-encoded genes (Kusumi et al., 2004; Munekage et al., 2004; Jarvis and Lopez-Juez, 2013; Kusumi and Iba, 2014; Pogson et al., 2015). The first step is activation of plastid DNA replication and plastid DNA synthesis. The second step known as the chloroplast “build-up” stage establishes the chloroplast genetic system, in which a nuclear-encoded plastid RNA polymerase (NEP) preferentially transcribes genes encoding plastid gene expression machinery that promotes the transcription and translation in chloroplasts (Hajdukiewicz et al., 1997). In the third stage, plastid genes, predominantly transcribed by a plastid-encoded plastid RNA polymerase (PEP), in combination with imported nuclear-encoded proteins, constitute the photosynthetic and metabolic machinery to control chloroplast development (Kanamaru et al., 1999). Taking rice (*Oryza sativa*) as an example, the plastid genome is about 135 kb composed of 34 RNA-coding genes and 120 protein-coding genes (Hiratsuka et al., 1989; Pfalz and Pfannschmidt, 2013). About 3,000 proteins function in chloroplasts, more than 95% of which are encoded by nuclear genes (Reumann et al., 2005), suggesting that chloroplast development is predominantly under control of nuclear genes. Therefore cloning and characterization of such nuclear genes should help elucidate the complex regulatory mechanisms of chloroplast development in plants.

Pentatricopeptide repeat (PPR) proteins, characterized by tandem arrays of degenerate PPR motifs, which are a type of commonly existing, evolutionarily widespread, plant protein segments composed of 35 canonical amino acids (Small and Peeters, 2000; Yin et al., 2013; Barkan and Small, 2014). According to the tandem motifs, PPR proteins have been classified into two subfamilies: P- and PLS-type. Based on the structure of C-terminal motifs, the latter was further classified into PLS, E, E+, and DYW subgroups (Lurin et al., 2004). Accumulating evidence shows that nuclear-encoded PPR proteins perform diverse functions in post-transcriptional modulation of gene transcripts, such as RNA processing (Kazama and Toriyama, 2003; Nakamura et al., 2003; Hattori et al., 2007), RNA editing (Hammani et al., 2009; Fujii and Small, 2011; Yap et al., 2015; Xie et al., 2016), RNA splicing (Tan et al., 2014; Hsieh et al., 2015; Yap et al., 2015), RNA stability (Beick et al., 2008; Tavares-Carreon et al., 2008; Hammani et al., 2016; Zoschke et al., 2016), RNA translation (Schmitz-Linneweber et al., 2005; Barkan et al., 2012; Zoschke et al., 2016), and RNA maturation (Wu et al., 2016; Zhou et al., 2017). Genetic evidence shows little or no redundancy of function between PPR proteins, although gene family has expanded in higher plants, with 450 members in Arabidopsis and 477 members in rice (Small and Peeters, 2000; Lurin et al., 2004; O’Toole et al., 2008). This evidence suggests that the functions of PPR proteins are highly diversified in higher plants.

Of the 477 PPR members in the rice genome, only a limited number has been cloned. Among them, RF5 and RF6 associate with GRP162 and hexokinase 6 to regulate mitochondrial RNA metabolism and fertility restoration, respectively (Hu et al., 2012; Huang et al., 2015). *OGR1* and *MPR25* encode a DYW motif-containing PPR protein and an E subgroup member of PPR, respectively, and both are involved in RNA editing in mitochondria (Kim et al., 2009; Toda et al., 2012). Their loss-of-function mutations caused retarded growth (Kim et al., 2009; Toda et al., 2012). In addition to the mitochondrially-localized PPR proteins listed above, previous studies also identified several chloroplast-targeted PPR members, including OsPPR1 (Gothandam et al., 2005), YSA (Su et al., 2012), OsV4 (Gong et al., 2014), WSL (Tan et al., 2014), ASL3 (Lin et al., 2015a), and OspTAC2 (Wang D. et al., 2016). A conspicuous feature of these rice mutants or their corresponding RNAi transgenics is the chlorophyll (Chl) deficient phenotype and abnormally developed chloroplasts at seedling stage, suggesting essential roles in chloroplast development at an early leaf growth stage. Due to lack of in-depth functional studies on these genes, only WSL has thus far been implicated in RNA splicing of chloroplast transcript *rpl2* (Tan et al., 2014). Additionally, OspTAC2 may act as the core subunit of the PEP complex based on studies of the Arabidopsis homolog pTAC2 (Wang D. et al., 2016). Despite significant advances, the roles of the PPR proteins in regulating post-transcriptional modification of organelle genes, especially chloroplast transcripts, remain obscure. It is expected that cloning and more in-depth studies of PPR proteins will help to decipher the regulatory network of post-transcriptional modulation of gene expression in rice.

Here, we identified rice Chl deficient mutant *wsl4* that develops striped leaves with decreased Chl contents and impaired chloroplast structures at the early leaf developmental stage. We show that *WSL4* encodes a novel P-family PPR protein that targets to the chloroplast nucleoid. Expression analyses suggested that WSL4 coordinates expression of many nuclear- and plastid-encoded genes involved in chloroplast development. We found that WSL4 could be involved in chloroplast development by affecting RNA splicing at an early stage of leaf development.

## Materials and Methods

## Plant Materials and Growth Conditions

The *wsl4* mutant was isolated from a ^60^Co-irradiated mutant pool of *japonica* cultivar RX69. The mutagenesis screen was performed as described previously (Wang L. et al., 2016; Zhou et al., 2016). A cross was made between the *wsl4* mutant and RX69 for preliminary genetic analysis and a large F_2_ segregation population was generated from *wsl4* × Yue13 (ssp. *indica*) for fine mapping. Plants were grown in a paddy field during the normal rice growing season in Beijing (39°54′N, summer season) or in a growth chamber with a 12 h photoperiod at 30/25°C (L30/D25). Seedlings for differential temperature studies were grown in a growth chamber with a 12 h photoperiod and constant 30°C (C30) and 20°C (C20).

### Chlorophyll Contents and Transmission Electron Microscopy (TEM) Analyses

Chlorophyll contents were measured according to a method described by Wu et al. (2007). Briefly, fresh leaves of WT and *wsl4* mutant plants were collected at the two-, three-, four-, and five-leaf stages, and then cut and marinated in 5 ml of 95% ethanol for 48 h in darkness. After centrifugation, the residual plant debris was removed. The supernatants were analyzed with a DU 800 UV/Vis Spectrophotometer (Beckman Coulter) at 665, 649, and 470 nm, respectively.

For TEM analyses, third leaves from WT and green and white sectors of *wsl4* mutant were cut into slices of 0.5 cm and three slices of each tissue were fixed in a solution of 2.5% glutaraldehyde in phosphate buffer at 4°C for 4 h, and incubated overnight at 4°C in 1% OsO4. The tissues were subsequently dehydrated in an ethanol series, infiltrated in a gradient series of epoxy resin, and finally embedded in Spurr’s medium prior to thin sectioning (50–80 nm). Samples were stained again and examined with a Hitachi H-7650 transmission electron microscope.

### Fine-Mapping of *WSL4* Locus

Sequence polymorphisms between Nipponbare (*japonica*) and 93-11 (*indica*) were identified from a public database^1^ and used to develop Indel markers for fine mapping. Primer pairs were designed with Primer Premier 3.0. Newly developed PCR-based molecular markers used in this study are listed in Supplementary Table S1. The PCR procedure was as follows: 94°C for 5 min, followed by 34 cycles of 94°C for 30 s, annealing for 30 s, 72°C for 30 s, and a final elongation step at 72°C for 7 min.

### Complementation Test and RNAi Suppression of WSL4

For complementation of the *wsl4* mutation a 5.7 kb WT genomic fragment (primer pairs PPR-G, Supplementary Table S1) containing a 2.2 kb upstream sequence, the entire coding region of *WSL4*, and a 2.0 kb downstream sequence was amplified from RX69 with Prime STAR HS DNA Polymerase (TaKaRa) and cloned into the binary vector pCAMBIA1305 to generate the vector pCAMBIA1305-G*WSL4*. This vector was introduced into *Agrobacterium tumefaciens* EHA105, which was then used to infect *wsl4* mutant *calli* (Hiei et al., 1994).

For RNAi test, the construct pCUbi1390-DFAD2 (ubiquitin promoter and a FAD2 intron inserted into pCAMBIA1390) was used as an RNAi vector (Stoutjesdijk et al., 2002; Wu et al., 2007). Both anti-sense and sense versions of a specific 332 bp fragment from the coding region of the *WSL4* 5′-end were amplified (primer pairs *WSL4*-RNAi-SacI-InF and *WSL4*-RNAi-BamHI-InR, Supplementary Table S1), and successively inserted into pCUbi1390-DFAD2, to form the RNAi construct pUbi-dsRNAiWSL4. The construct was then used to infect the *calli* produced from RX69. Transformation was conducted according to the method described above.

### Sequence and Phylogenetic Analysis

Gene prediction was performed using the GRAMENE database^2^. Homologous sequences of WSL4 were identified using the Blastp search program of the National Center for Biotechnology Information (NCBI^3^). A chloroplast transit peptide at the N-terminus of WSL4 was predicted by ChloroP^4^ and TargetP^5^. A phylogenetic tree was constructed using MEGA v4.1 software^6^ by the bootstrap method with 1,000 replicates. Multiple sequence alignments were conducted with BioEdit software (Borland Company).

### Subcellular Localization of WSL4 Protein

For subcellular localization of WSL4 and wsl4 proteins in rice the coding sequences of *WSL4* and *wsl4* were amplified using specific primer pairs (listed in Supplementary Table S1) and cloned into the transient expression vector pA7-GFP to generate the fusion genes *WSL4-GFP* and *wsl4-GFP* driven by the CaMV 35S promoter. All transient expression constructs were separately transformed into rice protoplasts and incubated in darkness at 28°C for 16 h before examination according to the protocols described previously (Chiu et al., 1996; Chen et al., 2006). Fluorescence of GFP in transformed protoplasts was visualized using a confocal laser scanning microscope (Leica TCS SP5).

### RT-PCR and Real-Time RT-PCR Analyses

Total RNA was extracted from flag leaves, culms, young panicles, and leaf sheaths at the booting stage, as well as shoot bases, leaves, and seedling roots using an RNA Prep Pure Plant kit (Tiangen Co., Beijing). Each RNA sample (about 3 μg) was reverse transcribed using primer Script I (TaKaRa) and an oligo(dT)18 primer for nuclear-encoded genes or random primers for plastid-encoded genes since mRNA from most plastid-encoded genes carried no poly-A tail. The RT-PCR procedure was as follows: 94°C for 5 min, followed by 33 cycles of 94°C for 30 s, annealing for 30 s, 72°C for 1 kb/s, and a final elongation step at 72°C for 7 min. The primer pairs are listed in Supplementary Table S1. For analysis of RNA splicing we sequenced full-length RT-PCR products with special primers flanking the introns (Supplementary Table S1). For Real-Time RT-PCR analysis of RNA splicing efficiency, specific primers (Supplementary Table S1) were designed for intron-exon (unspliced forms) and exon–exon (spliced forms) links of each gene (de Longevialle et al., 2007; Koprivova et al., 2010; Hsieh et al., 2015; Yap et al., 2015).

Real-Time RT-PCR was performed using a SYBR Premix Ex Taq^TM^ Kit (TaKaRa) on an ABI prism 7500 Real-Time PCR System. The program was as follows: initial polymerase activation for 30 s at 95°C followed by 40 cycles of 95°C for 5 s and 60°C for 34 s. The 2^-ΔΔCT^ method was used to analyze relative transcript levels of genes (Livak and Schmittgen, 2001). The primer sequences for qRT-PCR are listed in Supplementary Table S1. The rice *Ubiquitin* gene (*LOC_Os03g13170*) was used as a reference in qRT–PCR (primer pairs *Ubq*).

### Northern Blot Analyses

Total RNA from leaves (L3-3) at the seedling stage was isolated using *Trans*Zol Up (TransGen) following recommendations by the manufacturer. About 2 μg of RNA was fractionated in a 1.2% (w/v) denaturing formaldehyde agarose gel and transferred onto Hybond N+ nylon membranes (GE Healthcare Biosciences). Digoxigenin-labeled probes were obtained by PCR using a DIG Northern Starter Kit (Roche). The primers are listed in Supplementary Table S1. Prehybridization of the membrane was carried out for 1 h at 68°C in PerfectHyb Plus Hybridization Buffer (SIGMA). Hybridization was performed overnight in the same buffer at 68°C. Signal was detected using the DIG Wash and Block Buffer Set (Roche), and eventually read in an imaging system (Tanon 5200).

### Protein Extraction and Western Blot Analyses

Fresh leaves (L3-3) were standardized by fresh weight and ground into fine powder. Total plant proteins were extracted with an appropriate volume (2 mL g^-1^) of NB1 buffer [50 mM Tris-MES, 1 mM MgCl_2_, 0.5 M sucrose, 10 mM EDTA, 5 mM DTT, and protease inhibitor cocktail (Roche, Cat. No. 11 836-153-001; the used concentration was 1 tablet for a volume of 50 ml solution), pH 8.0]. The protein samples were resolved in 10% SDS–polyacrylamide gel electrophoresis (PAGE) gels, and then transferred onto PVDF membranes (0.45 μm, Millipore), followed by incubation with antibodies. Signals were detected using a Luminata^TM^ Forte Western HRP Substrate (Millipore) and visualized by an imaging system (ChemiDocTMX- RS; Bio-Rad). The polyclonal antibodies used in this study were obtained from BGI^7^. The intensities of chloroplast proteins were quantified by “ImageJ” software.

### Statistical Analysis

All statistical analyses were performed using Student’s *t*-test. The number of biological replicates (*n*) in each experiment is indicated in the corresponding figure legends. Values were considered statistically significant at *P* < 0.05, and very significant at *P* < 0.01.

## Results

### Phenotypic Characterization of the *wsl4* Mutant

**FIGURE 1:**
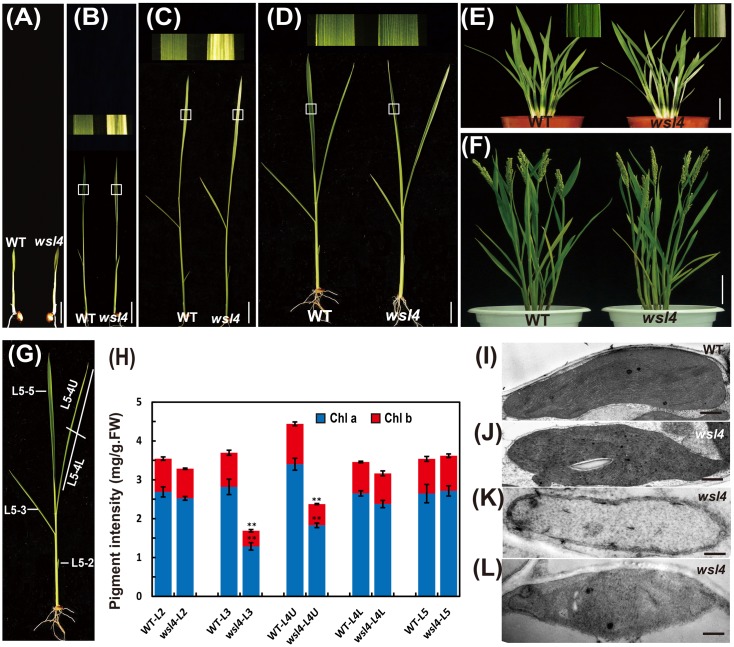
Phenotypic comparison of WT and *wsl4* mutant plants. Comparisons of leaves of WT and *wsl4* mutant plants grown in a growth chamber with a 12 h photoperiod at 30°C/25 °C (L30/D25) at the two- **(A)**, three- **(B)**, four- **(C)**, and five- leaf **(D)** stages. The insets represent magnified views of the selected areas in **(B–D)**. Bars, 0.5 cm. **(E,F)** Leaves of WT and *wsl4* mutant plants grown in field at the tillering stage **(E)** and after heading stage **(F)**. Note the white-striped leaves from the tillers of the *wsl4* mutant at the tillering stage. Bars, 5 cm **(E)**; 10 cm in **(F)**. **(G)** A rice shoot with fully emerged fifth leaf at the five-leaf stage. L5-2, L5-3, L5-4, and L5-5 represent the second, third, fourth, and fifth leaf at the five-leaf stage, respectively. **(H)** Measurements of chlorophyll a and b contents of all leaves (L5-2, L5-3, L5-4U, L5-4L, and L5-5) from five-leaf stage WT and *wsl4* mutant plants grown in a growth chamber at L30/D25. L5-4U represents the upper half of the fourth leaf, and L5-4L represents the basal half of the fourth leaf. Values are means ± SD from three independent repeats. Student’s *t*-test: ^∗^*P* < 0.05; ^∗∗^*P* < 0.01. Chl a, chlorophyll a, Chl b, chlorophyll b, FW, fresh weight. **(I–L)** Electron micrographs of chloroplasts from L3-3 of WT **(I)** and *wsl4* mutant **(J–L)** plants grown in a growth chamber at L30/D25. Chloroplasts from the WT plants **(I)** and chloroplasts from the green portions of the *wsl4* mutant **(J)** plants have abundant and well-ordered thylakoids and stacked membranes, whereas chloroplasts from white striped sectors of the *wsl4* mutant plants had almost no normal stacked membrane structures **(K,L)**. L3-3 indicates the third leaf at the three-leaf stage. Bars, 0.5 μm **(I,J,L)**; 0.2 μm in **(K)**.

To characterize the *wsl4* mutant in detail, we conducted a time-course examination of the mutant phenotype from the two to five-leaf stages. The leaves of seedlings at the 3–4 leaf stage exhibited a bleached appearance with the most extreme symptoms observed in the third leaf and distal half of the fourth leaf (**Figures 1A–C**). All leaves after the 5-leaf stage were as green as WT plants (**Figure 1D**). Young leaves on newly emerging tillers of the *wsl4* mutant also exhibited the green/white leaf color defect (**Figure 1E**). No difference was observed between WT and *wsl4* mutant after the heading (**Figure 1F**). Consistent with these observations, levels of Chl a and b in striped leaves of *wsl4* plants were much lower than those in WT (**Figures 1G,H**) Compared with WT plants, *wsl4* mutants showed no statistically significant differences in plant height, number of tillers, number of branches per panicle, number of spikelets per panicle, and 1000-grain weight (**Table 1**).

**Table 1:** Statistical analyses of major agronomic traits between WT and the *wsl4* mutant.

Genotype	Plant height (cm)	Number of tillers per plant	Branch numbers per panicle	Number of spikelets per panicle	1000-grain weight (g)
Wild type	84.4 ± 2.2	7.2 ± 0.8	12.6 ± 0.7	147.0 ± 17.7	25.3 ± 0.1
*wsl4*	84.9 ± 2.9	7.7 ± 0.5	13.1 ± 0.6	138.0 ± 15.1	25.5 ± 0.1


*Value is mean ± SD from 30 plants. Student’s *t*-test (*n* = 10) at significant level of 0.05.*

**FIGURE 2:**
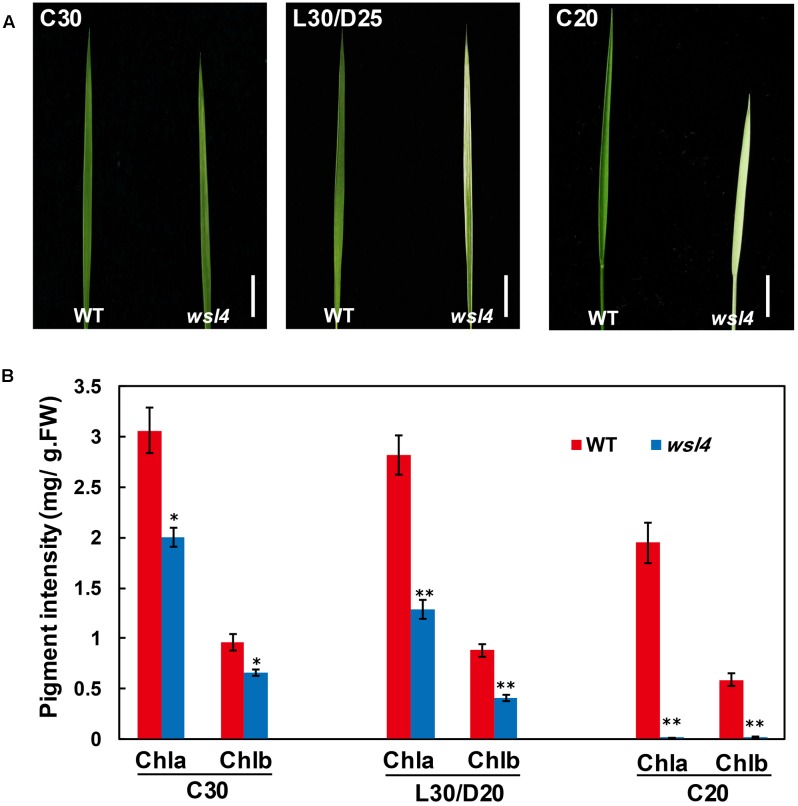
Response of the wsl4 mutant to temperature. **(A)** WT and *wsl4* mutant seedlings grown in a growth chamber with 12 h photoperiod and 30°C (C30), 30/25°C (L30/D25), and 20°C (C20). Bars, 0.5 cm. **(B)** Chl a and Chl b contents in L3-3 from WT and *wsl4* mutant seedlings (*n* = 6) grown in a growth chamber with the same conditions as **(A)**. Student’s *t*-test: ^∗^*P* < 0.05; ^∗∗^*P* < 0.01.

In contrast to the moderate striped phenotype and Chl accumulations under optimum temperature (L30/D25), the *wsl4* mutant exhibited less color deficiency and accumulated much higher Chl contents when grown at a constant 30°C (C30) (**Figure 2**). At a constant 20°C the *wsl4* mutant showed more extreme symptoms and Chl content that were barely detectable (**Figure 2**). Our results show that the *wsl4* mutant is sensitive to low temperatures.

### The *wsl4* Mutant Has Disrupted Chloroplast Development

To determine whether the color deficiency resulting from the decreased photosynthetic pigment accumulation in the *wsl4* mutant were associated with ultrastructural damages in chloroplasts, we compared the ultrastructural features of chloroplasts from WT and *wsl4* mutant leaves using TEM. As shown in **Figures 1I–L**, the lamellar structures of WT chloroplasts were well developed and were equipped with normally stacked grana and thylakoid membranes (**Figure 1I**). While green sectors of *wsl4* mutant seedlings were still able to develop normal chloroplasts (**Figure 1J**), the white striped regions had abnormal chloroplasts that lacked organized lamellar structures (**Figures 1K,L**). Thus the stripe leaf phenotype and the reduced Chl contents in the *wsl4* mutant leaves were probably due to developmentally defective chloroplasts.

### The *WSL4* Gene Encodes a Novel P-Family PPR Protein

**FIGURE 3:**
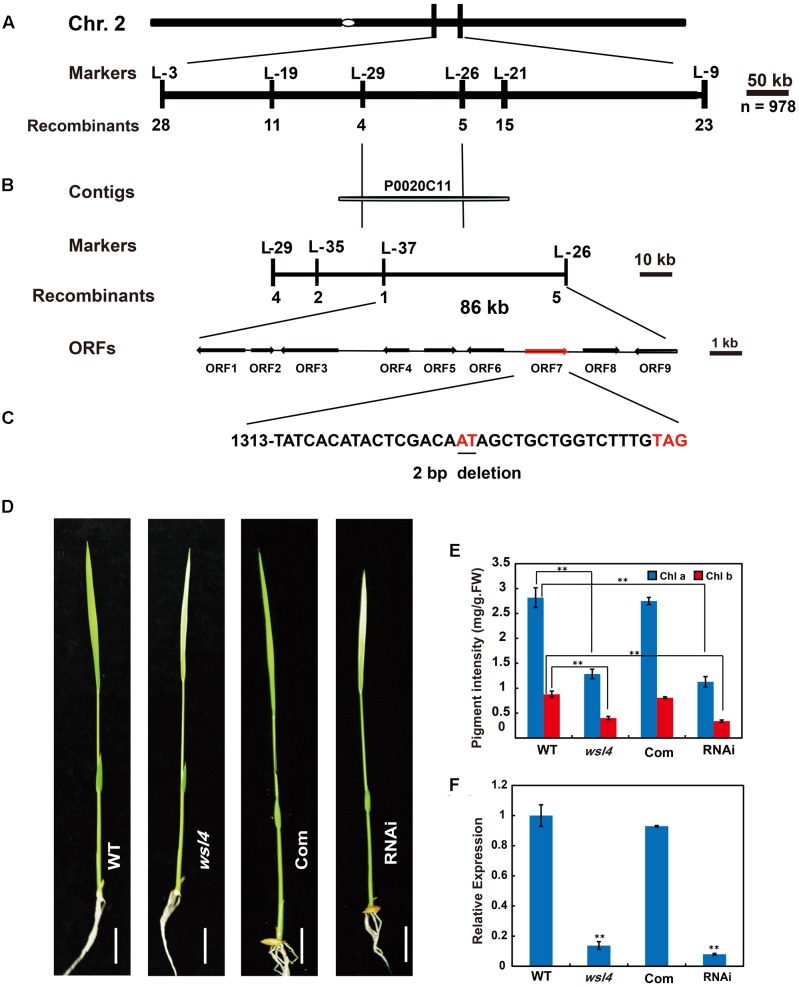
Map-based cloning of the *WSL4* gene. **(A)** The *WSL4* locus was initially mapped on the long arm of chromosome 2 between the insertion/deletion markers L-3 and L-9. **(B)** The *WSL4* locus was ultimately narrowed to an 86-kb region between the insertion/deletion markers L-37 and L-26 on BAC clone P0020C11 using 978 F_2_ homozygous mutant background plants. Nine open reading frames (ORFs) were predicted in the region. **(C)** Comparison with WT cDNA sequence revealed a 2 bp deletion (*underlined*) in ORF7, generating a premature stop codon. **(D)** Phenotypic comparison at the three-leaf stage of WT, *wsl4* mutant, complemented T_1_ (Com), and RNAi T_1_ plants grown in a growth chamber at L30/D25. Bars, 0.5 cm. **(E)** Chlorophyll contents of L3-3 from plants corresponding to **(D)**. L3-3 indicates the third leaf at the three-leaf stage. Values are means ± SD from three independent repeats. Student’s *t*-test: ^∗^*P* < 0.05; ^∗∗^*P* < 0.01. Chl a, chlorophyll a, Chl b, chlorophyll b, FW, fresh weight. **(F)** Real-time RT-PCR analyses of expression levels of the *WSL4* gene in L3-3 of plants corresponding to **(D)**. The *ubiquitin* gene was used as an internal control. Data are means ± SD of three repeats. Student’s *t*-test: ^∗^*P* < 0.05; ^∗∗^*P* < 0.01.

Genetic analyses of reciprocal crosses between the WT and *wsl4* mutant showed that the *wsl4* phenotype was inherited by a single recessive nuclear mutation (**Table 2**). Using the F_2_ population derived from the *wsl4*/Yue13 cross, the *wsl4* locus was initially mapped to the long arm of rice chromosome 2, between the insertion/deletion markers L-3 and L-9 (**Figure 3A**). Based on 978 F_2_ recessive homozygous *wsl4* background individuals, the *WSL4* locus was ultimately narrowed down to an 86-kb genomic region flanked by the insertion/deletion markers L-37 and L-26 on the BAC clone P0020C11, in which nine putative open reading frames (ORFs) were predicted (**Figure 3B**). Comparison with WT cDNA sequences revealed a 2 bp deletion of positions 1,330 and 1,331 bp from the ATG start codon in the 7th ORF (*LOC_Os02g35750*) (**Figure 3C**), generating a premature stop codon. Sequence comparison between genomic and cDNA showed that the *WSL4* gene contained a single exon, which was predicted to encode a 554 amino acid residue polypeptide with a calculated molecular mass of approximately 60 kDa. By contrast, the *wsl4* gene, if translated, would encode a truncated protein, named wsl4, and missing the C-terminal 111 amino acid residues, but with five added unrelated amino acid residues resulting from translation of the frame-shift (**Supplementary Figure S1**).

**Table 2:** Segregation of mutant phenotypes in reciprocal crosses between WT and the *wsl4* mutant.

Cross	Normal	Striped	χ^2^_3:1_^a^
wild type/*wsl4* F_2_	388	128	0.010
*wsl4*/wild type F_2_	363	123	0.025


*^a^Value for significance at *P* = 0.05 and χ^*2*^_*3:1*_ for 1 *df* is 3.84.*

To test whether the 2 bp deletion conferred the *wsl4* mutant phenotype, a 5.7 kb WT genomic fragment harboring the transcriptional regulation elements and putative full-length coding sequence of *LOC_Os02g35750* was introduced into the *wsl4* mutant. All six positive transgenic lines exhibited green leaves, and had comparable levels of Chl a and b to the WT (**Figures 3D,E**). To further confirm that disruption of *LOC_Os02g35750* function was responsible for the *wsl4* mutant phenotype, we generated RNAi transgenic lines in WT background and obtained four independent transgenic lines, that phenocopied the *wsl4* mutant with respect to leaf color and Chl contents (**Figures 3D,E**). Thus *LOC_Os02g35750* corresponds to the *WSL4* gene.

**FIGURE 4:**
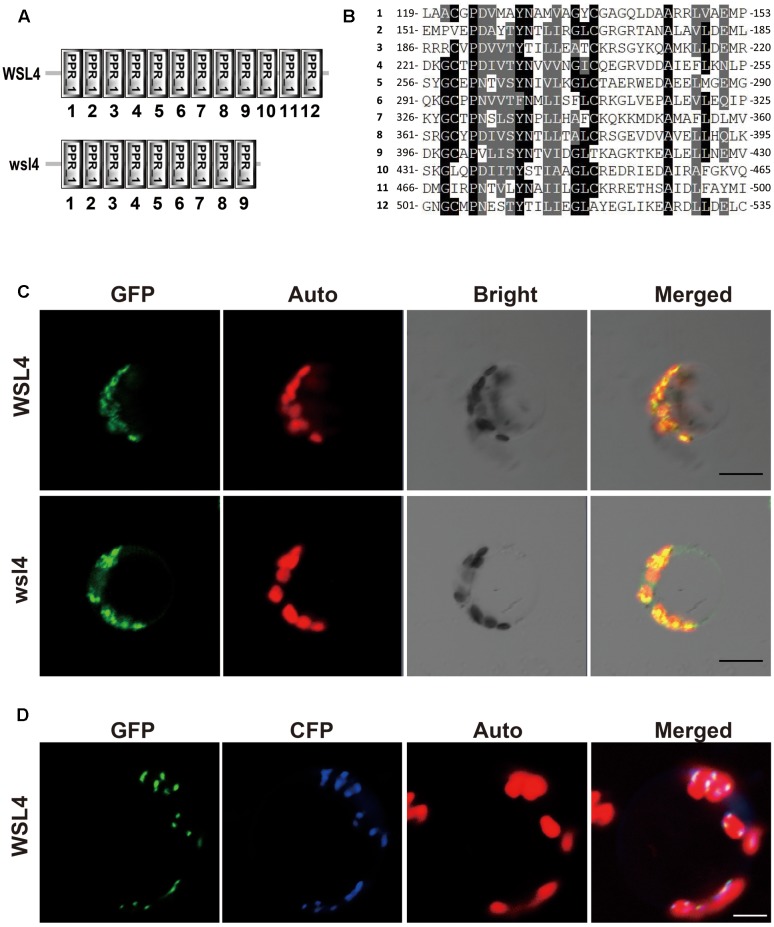
Sequence analysis and subcellular localization of the WSL4 protein. **(A)** WSL4 protein has 12 PPR motifs, whereas the *wsl4* mutant protein contains only 9. **(B)** The 12 PPR motifs of WSL4. **(C)** Transient expression of WSL4–GFP and wsl4–GFP fusion proteins in rice protoplasts. **(D)** Colocalization of WSL4 (GFP) and PEND (CFP) within chloroplast nucleoids. GFP, GFP signals of WSL4 fusions; CFP, CFP signals of chloroplast nucleoid marker PEND; Auto, Chl autofluorescence; Merged, Merged images of GFP, CFP, and Auto. Bars, 5 μm.

An analysis of WSL4 protein in the Pfam database (Finn et al., 2010) showed that it contained a tandem repeat of 12 PPR motifs and belonged to a member of the P subfamily (**Figure 4A**). The 12 PPR motifs in WSL4 protein were sequentially arranged, with an average 31.5% sequence identity among them (**Figure 4B**). The 2 bp deletion in *wsl4* mutant occurred at the middle of the 10th PPR motif site, resulting in loss of the last three PPR repeats (**Figure 4A** and **Supplementary Figure S1**). The fact that *wsl4* mutant exhibited the same or a slightly weaker phenotype (**Figures 3D–F**) and splicing defects (see below) as the *WSL4* knock-down transgenic lines suggests that the 2 bp deletion in *wsl4* mutant causes the partial loss-of-function mutation.

A BLAST search of the NCBI database showed that *WSL4* is a single-copy gene in the rice genome, and homologs can be found in many other plant genomes. We performed phylogenetic analyses of WSL4 and these homologs to determine their evolutionary relatedness. The WSL4 protein shared high sequence homology with homologs in *Brachypodium distachyon*, *Hordeum vulgare*, *Setaria italic*, *Zea mays*, *Sorghum bicolor*, and *Aegilops tauschii* (**Supplementary Figures S2**, **S3**); however, the functions of these genes including the Arabidopsis homolog were unknown. The WSL4 protein thus defined a new member of the P-superfamily of PPR proteins.

### Subcellular Localization of WSL4 Protein

The prediction of a chloroplast transit peptide at the N-terminus of WSL4 by ChloroP^8^ and TargetP^9^, together with the Chl-deficient phenotype of *wsl4* mutant, suggests that WSL4 is localized to chloroplasts. To determine its actual localization, we transiently expressed *p35S:WSL4-GFP* in rice protoplasts. As shown in **Figure 4C** confocal microscopy observations showed that WSL4-GFP was localized to punctate compartments associated with Chl autofluorescence. Moreover, we noted that wsl4-GFP was still targeted to the chloroplast, suggesting that the 111 missing amino acid residues at the C-terminus of WSL4 were not necessary for chloroplast localization (**Figure 4C**). To further determine the nature of these small dot-like structures, we coexpressed WSL4-GFP and CFP-tagged plastid binding protein PEND-CFP known to be a fluorescent marker protein for plastid nucleoids (Terasawa and Sato, 2005). As expected, the GFP signals colocalized with the CFP signals, indicating that WSL4 was localized to the chloroplast nucleoids (**Figure 4D**). These results show that WSL is indeed a chloroplast nucleoid-localized protein.

### Expression Analyses of *WSL4* and Genes Associated with Chloroplast Development

**FIGURE 5:**
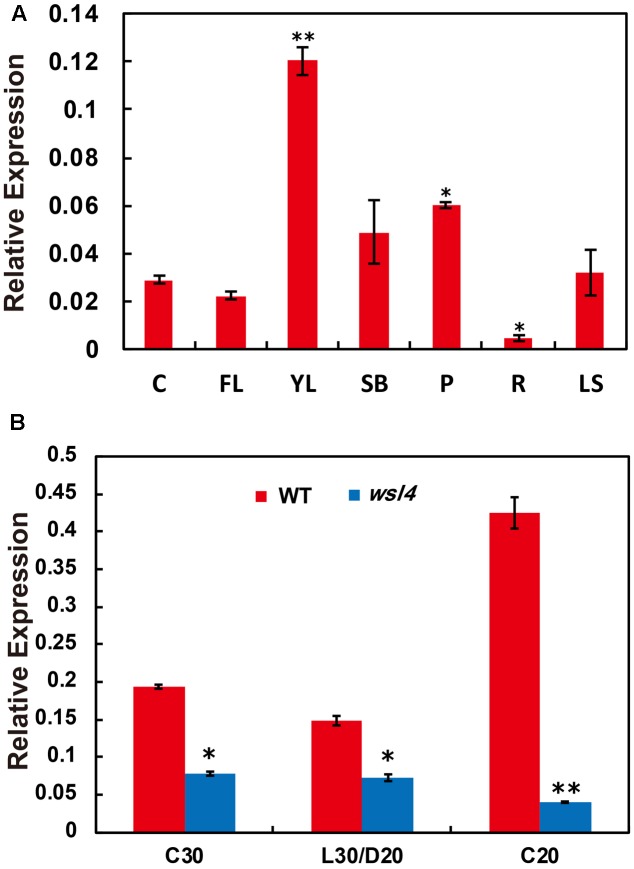
Expression analyses of *WSL4*, chloroplast-encoded, and nuclear-encoded genes as well as immunoblot analyses of plastidic proteins. **(A)** Relative expression of *WSL4* in culms (C), flag leaf (FL), young panicles (P), and leaf sheaths (LS) from field-grown WT plants grown in the field at the booting stage, as well as young leaves (YL), shoot bases (SB), and young roots (R) from WT seedlings grown in a growth chamber at L30/D25. For *t*-test, values were compared with expression level of *WSL4* in culms. Student’s *t*-test: ^∗^*P* < 0.05; ^∗∗^*P* < 0.01. **(B)** qRT-PCR analyses of *WSL4* transcript in L3-3 from WT and *wsl4* mutant seedlings grown in a growth chamber with a 12 h photoperiod at C30, L30/D25, and C20. Values are means ± SD of three replicates. Student’s *t*-test: ^∗^*P* < 0.05; ^∗∗^*P* < 0.01.

A survey of the rice expression profile database^10^^,^^11^ revealed that *WSL4* was constitutively expressed in all tissues examined, with the strongest expression in the young leaves. For validation, expression analyses performed by quantitative RT-PCR (qRT-PCR) using RNA samples prepared from culms (C), flag leaves (FL), young leaves (YL), shoot bases (SB), young panicles (P), young roots (R), and leaf sheaths (LS) showed that the abundance of *WSL4* transcript was significantly higher in young leaves compared to other tissues (**Figure 5A**). These expression data together with the observed *wsl4* phenotype support the hypothesis that *WSL4* has an important role at chloroplast development stage during the early leaf development in rice. Interestingly, accumulation of *WSL4* transcript was enhanced under low temperature conditions (**Figure 5B**).

**FIGURE 6:**
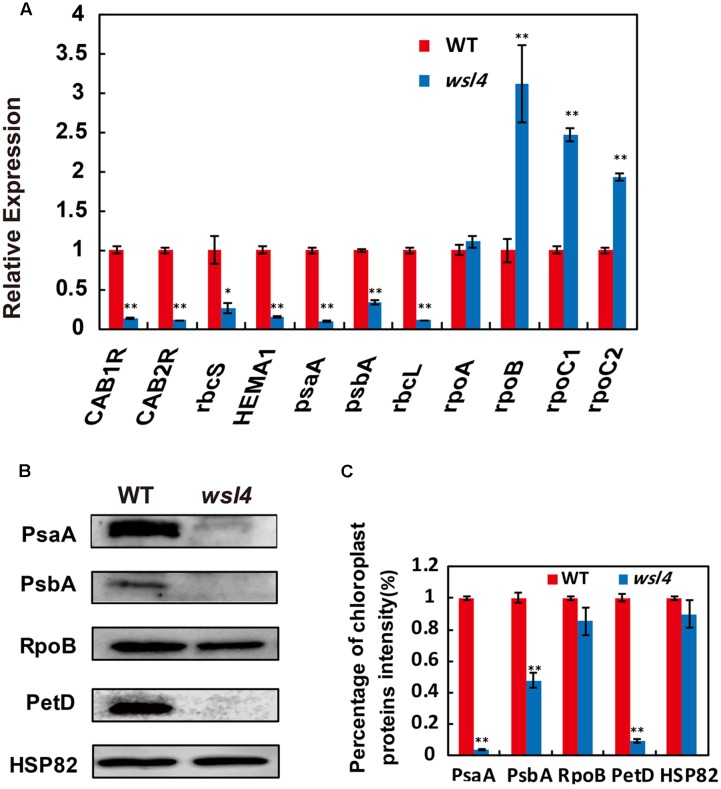
Expression analyses of chloroplast-encoded, and nuclear-encoded genes as well as immunoblot analyses of plastidic proteins. **(A)** qRT-PCR analyses of genes associated with chloroplast development in WT and *wsl4*. Total RNA was extracted from L3-3 of the corresponding plants grown in a growth chamber at L30/D25. **(B)** Immunoblot analyses of photosynthetic proteins in WT and the *wsl4* mutant. Total proteins were extracted from L3-3 of WT and *wsl4* mutant plants grown in a growth chamber at L30/D25. L3-3 indicates the third leaf at the three-leaf stage. HSP82 was used as an internal control. **(C)** Quantification of the band intensity of photosynthetic proteins in the *wsl4* mutant compared- to WT corresponding to **(B)**. Values are means ± SD of three replicates. Student’s *t*-test: ^∗^*P* < 0.05, ^∗∗^*P* < 0.01.

Multiple chloroplast-associated genes were investigated to further determine whether the defective chloroplast development was associated with the altered gene expression. As shown in **Figure 6A**, key photosynthesis-associated nuclear genes (*cab1R*, *cab2R*, and *rbcS*) were significantly down-regulated in *wsl4* mutant, suggesting that photosynthesis in *wsl4* mutant was compromised. Plastidic genes can be grouped into three types. Class I genes are mainly transcribed by PEP, whereas Class III genes are absolutely transcribed by NEP. In contrast, NEP functions cooperatively with PEP to regulate the transcription of Class II genes (Hedtke et al., 1997; Yu et al., 2014). We next examined expression levels of Classes I and plastid genes. Expression of PEP-dependent Class I genes (e.g., *psaA*, *psbA*, and *rbcL*) was significantly down-regulated in the *wsl4* mutant, whereas expression of NEP-dependent Class III genes (e.g., *rpoB*, *rpoC1*, and *rpoC2*) was significantly up-regulated (**Figure 6A**). These results suggest that PEP complex activity was also compromised in the *wsl4* mutant as well. Consistent with the reduced Chl content, expression of Chl synthesis genes *CHLD*, *CHLI*, *CHLH*, and *PORA*, but not *YGL1*, was significantly inhibited in *wsl4* mutant (**Supplementary Figure S4**). We therefore propose that *WSL4* might coordinate the expression of numerous nuclear- and plastid-encoded genes to regulate chloroplast development at the early leaf development stage.

We finally assessed the accumulation of core subunits of photosynthetic enzyme complexes in WT and the *wsl4* mutant, including photosystem I (PsaA and PsaB) and photosystem II (PsbA and PsbD) subunits, RNA polymerase subunits (RpoB), ATP synthase CF1 β subunit (AtpB), and cytochrome b6f (PetD). All assayed proteins in bleached tissues, except RpoB, were barely detectable in the *wsl4* mutant compared to WT (**Figures 6B,C**).

### WSL4 Affects the Editing Efficiency of *rpoB* Gene

**FIGURE 7:**
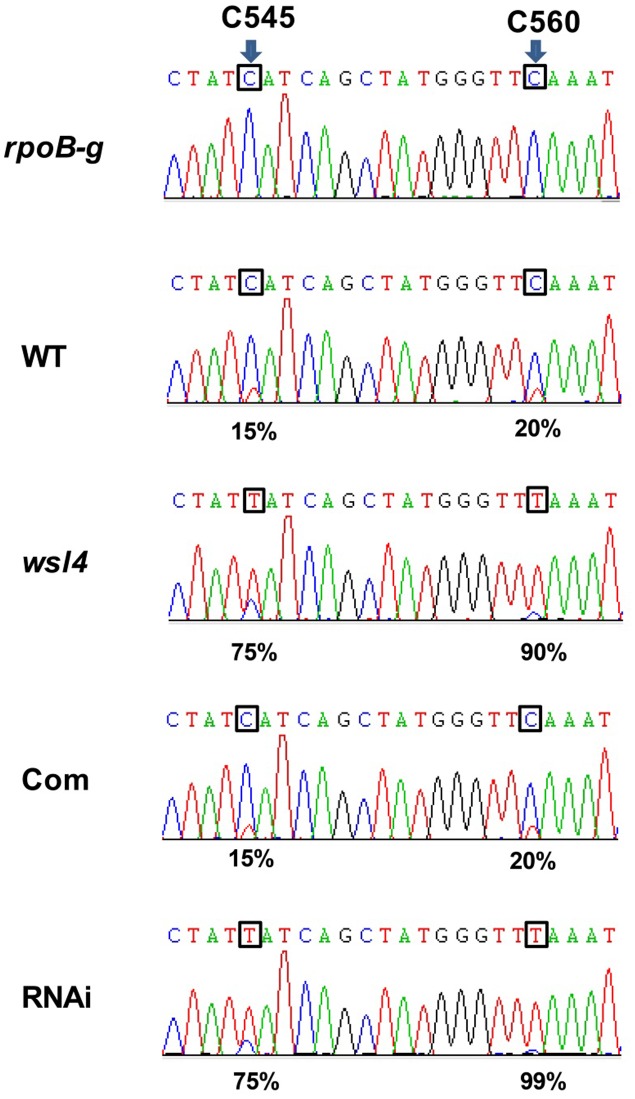
Editing efficiencies of *rpoB* genes in WT and the *wsl4* mutant. RT-PCR products of *rpoB* transcripts from L3-3 of WT, *wsl4* mutant (white striped sector), complemented (Com), and RNAi (white striped sector) plants grown in a growth chamber at L30/D25 were sequenced. In *wsl4* mutant and RNAi plants, the editing efficiency of *rpoB* at C545 and C560 were significantly increased compared to WT and Com. The top panel corresponds to the genomic nucleotide sequence. Green, black, red, and blue peak represent A, G, T, and C, respectively. Arrowheads indicate the editing sites and their position in the corresponding chloroplast cDNA strand. L3-3 indicates the third leaf at the three-leaf stage.

Accumulating evidence showed that a large group of nuclear-encoded PPR proteins required for RNA editing, splicing, stability, and translation were critical for chloroplast development (Hattori et al., 2007; Beick et al., 2008; Schmitz-Linneweber and Small, 2008; Barkan et al., 2012; Tan et al., 2014; Hsieh et al., 2015; Hammani et al., 2016; Xie et al., 2016). Firstly, we determined whether loss of WSL4 function affected editing at 21 identified RNA editing sites in chloroplast RNA (Corneille et al., 2000). The results showed that the editing efficiency of *rpoB* at C545 and C560 exhibited a significant increase in *wsl4* mutant compared with WT (**Figure 7**), while the rest 11 genes and the corresponding 19 editing sites were normally edited in *wsl4* mutant (**Supplementary Figure S5**). Furthermore, we analyzed the editing efficiency of *rpoB* at C545 and C560 in complemented and RNAi transgenic plants. As expected, the editing efficiency of *rpoB* at C545 and C560 showed a markedly reduction in complemented plant, while the RNAi plant mimicked a similar increase in RNA editing as the *wsl4* mutant (**Figure 7**). These data further suggest that the mutation in *WSL4* affects the editing efficiency of *rpoB* gene.

### The *wsl4* Mutant Is Defective in the Splicing of Chloroplast Group II Introns

**FIGURE 8:**
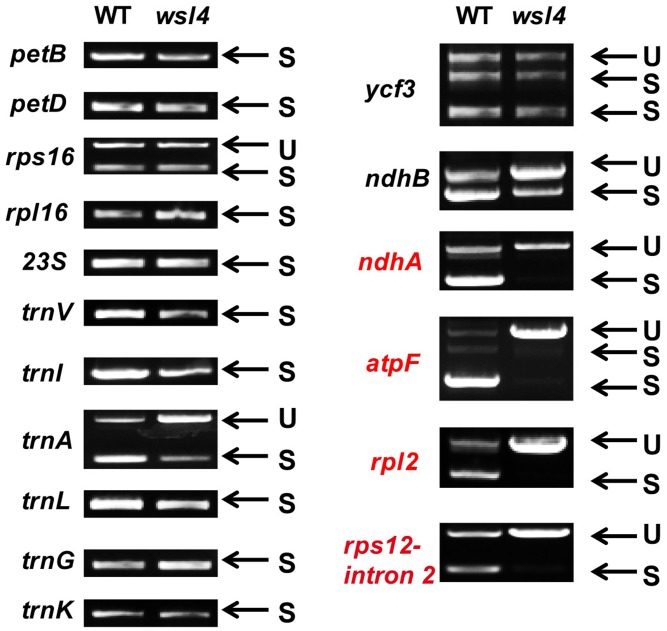
Splicing analyses of rice chloroplast transcripts in WT and *wsl4* mutant (white striped sectors). Gene transcripts are labeled at the left. Spliced (S) and unspliced (U) transcripts are shown at the right. RNA was extracted from L3-3 of WT and *wsl4* mutant plants grown in a growth chamber at L30/D25. L3-3 indicates the third leaf at the three-leaf stage.

**FIGURE 9:**
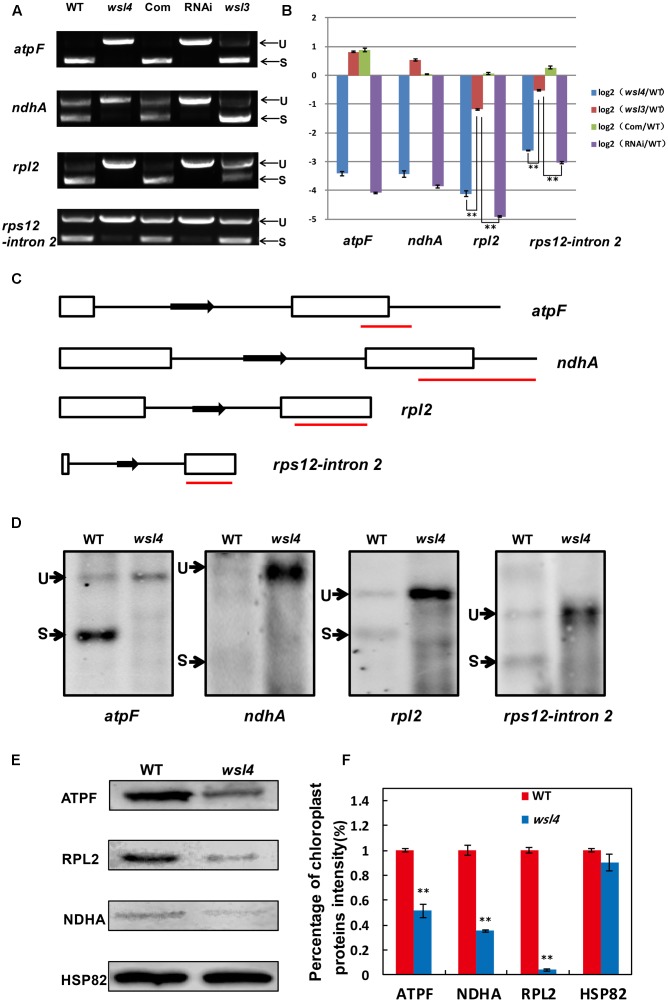
Splicing analyses of four chloroplast group II introns in WT and *wsl4* mutant. **(A)** RT-PCR analyses of *atpF*, *ndhA*, *rpl2*, and *rps12* transcripts in WT, *wsl4* mutant (white striped sector), complemented (Com), RNAi (white striped sector), and a control *wsl3* mutant with a transitory variegation phenotype. RT–PCR was performed with RNA extracted from L3-3 of the corresponding plants grown in a growth chamber at L30/D25. L3-3 indicates the third leaf at the three-leaf stage. **(B)** Quantitative RT-PCR analyses of *atpF*, *ndhA*, *rpl2*, and *rps12* transcripts in WT, *wsl4* mutant (white striped sector), Com, RNAi (white striped sector), and the *wsl3* mutant plants grown in a growth chamber at L30/D25. Histograms show log2 ratios of spliced to unspliced RNA in *wsl4*, *wsl3*, Com, and RNAi plants as compared with WT. Values are means ± SD of three replicates. **(C)** Sketch map of the *atpF*, *ndhA*, *rpl2*, and *rps12* transcripts. The red lines represent the probes used in **(D)**. **(D)** RNA gel blot assays of *atpF*, *ndhA*, *rpl2*, and *rps12* splicing in WT and *wsl4* mutant. Positions of spliced (S) and unspliced (U) transcripts are shown at the left. RNA was extracted from L3-3 of WT and *wsl4* mutant plants grown in a growth chamber at L30/D25. L3-3 indicates the third leaf at the three-leaf stage. **(E)** Immunoblot analyses of ATPF, NDHA, and RPL2 in WT and *wsl4* mutant at the three-leaf stage. Total proteins were extracted from L3-3 of WT and *wsl4* mutant plants grown in a growth chamber at L30/D25. L3-3 indicates the third leaf at the three-leaf stage. HSP82 was used as an internal control. **(F)** Quantification of the band intensity of ATPF, NDHA, and RPL2 in *wsl4* mutant compared to WT corresponding to **(E)**. Data are means ± SD of three repeats. Student’s *t*-test: ^∗^*P* < 0.05; ^∗∗^*P* < 0.01.

Accumulating evidence shows that a large group of nuclear-encoded PPR proteins required for RNA editing, splicing, stability, maturation, and translation are critical for chloroplast development (Hattori et al., 2007; Beick et al., 2008; Barkan et al., 2012; Tan et al., 2014; Hsieh et al., 2015; Hammani et al., 2016; Wu et al., 2016; Xie et al., 2016; Zoschke et al., 2016; Zhou et al., 2017). To determine whether loss of *WSL4* function affected RNA splicing of chloroplast genes containing 17 group II introns and one group I intron (Hiratsuka et al., 1989), we carried out RT-PCR analyses using primers situated in exons flanking intron 1, and then compared the lengths of the amplified products between WT and *wsl4* mutant plants. Four chloroplast transcripts containing group II introns were spliced with much reduced efficiency in *wsl4* mutant compared to WT (**Figures 8**, **9A**). The splicing defects were largely rescued in complemented transgenic plants and were mimicked in RNAi transgenic plants (**Figure 9A**). As a control, the splicing efficiencies of *atpF, ndhA, rpl2*, and *rps12* intron 2 were not significantly affected in the *wsl3* mutant (**Figure 9A**), a rice chloroplast translation-defective mutant lacking an essential peripheral subunit of the PEP complex (Wang L. et al., 2016). Splicing of other chloroplast transcripts was not significantly impeded in *wsl4* mutant (**Figure 8**).

Quantitative RT-PCR was performed to quantify spliced (using primers situated in exons 1 and 2) and unspliced transcripts (using primers situated in exon 1 and intron 1) to further determine the extent of the splicing defects, Compared with *wsl3*, *wsl4* and RNAi transgenic plants showed strong splicing defects in *atpF*, *ndhA*, *rpl2*, and *rps12* transcripts (**Figure 9B**). Consistent with phenotypic expression, the splicing defects of *atpF*, *ndhA*, *rpl2*, and *rps12* in *wsl4* mutant appeared to be worse in plants frown at 20°C (**Supplementary Figure S6**) suggesting that loss of WSL4 function caused the temperature-sensitive leaf bleaching and splicing defects at the early leaf developmental stage. The defective splicing of these four chloroplast transcripts was confirmed by RNA gel blot hybridizations using specific probes (**Figures 9C,D**). As a means of further investigating the effects of impaired splicing of *atpF*, *ndhA*, and *rpl2* transcripts on post-processing, western blots were performed to examine protein accumulations of atpF, ndhA, and rpl2 in *wsl4* mutant. As shown in **Figures 9E,F**, atpF, ndhA, and rpl2 were present at lower levels in *wsl4* mutant compared with WT. We propose that *WSL4* functions in the splicing of chloroplast group II introns.

## Discussion

Previous studies isolated and characterized numerous rice leaf color mutants. Based on phenotype such mutants have been subdivided into various classes, including albino, chlorina, stripe, virescent, and zebra^12^ (Jung et al., 2003). In this study, we isolated a rice stripe leaf mutant named *wsl4*. In contrast to mutants exhibiting Chl deficient phenotypes throughout plant development, such as *ygl1* and *vyl* (Wu et al., 2007; Dong et al., 2013), *wsl4* is characterized by a transient variegated phenotype during early seedling leaf and tiller development (**Figure 1**). Why *wsl4* and some other mutants exhibit a stage-specific bleaching phenotype is an intriguing question. It is possible that *WSL4* is not required at later leaf developmental stages when other genes compensate for its function in chloroplast development. Indeed, *WSL4* is actually expressed at lower levels in more mature leaves such as flag leaves than in young leaves (**Figure 5A**). The *ysa* mutant likewise exhibits a seedling-specific albino phenotype, and the expression level of *YSA*, encoding a PPR protein, decreases with leaf development (Su et al., 2012).

The bleached tissue sectors in the *wsl4* mutant stretch longitudinally (striping), but the stripes do not extend along the entire leaf (**Figures 1**, **2**), suggesting that other factors might affect the expression of the *wsl4* phenotype. The white tissue sections in the *wsl4* mutant are devoid of normal chloroplasts and show significantly reduced expression of genes associated with Chl synthesis and chloroplast development. Low temperature is a key factor affecting chloroplast gene expression and particularly in exacerbating chloroplast translation defects caused by various mutations, such as ribosomal proteins (Gong et al., 2013; Song et al., 2014; Lin et al., 2015b) and RNA binding proteins (Gong et al., 2014; Tan et al., 2014). Our results show that *wsl4* is a low temperature-sensitive mutant (**Figure 2**). Furthermore expression of *WSL4* was increased at lower temperatures (**Figure 5B**). These results may be a reason for low temperature dependency of *wsl4* phenotype. Similar low temperature dependency phenotypes have also been observed in several previously reported mutants, such as *v1*, *v2*, *v3*, and *st1* (Sugimoto et al., 2007; Yoo et al., 2009; Kusumi et al., 2011).

PEP-dependent genes (e.g., *psaA*, *psbA*, and *rbcL*) and nuclear genes associated with photosynthesis (e.g., *cab1R*, *cab2R*, and *rbcS*) were significantly down-regulated in the *wsl4* mutant, whereas expression levels of NEP-dependent genes were increased (**Figure 6**). These results together with decreased accumulation of the main photosynthetic protein (**Figure 6**) and the Chl deficient phenotype (**Figure 1**) suggest that PEP function may be impaired in *wsl4* mutant. Similar phenotypes were observed in several previously reported PEP-deficient mutants including *ptac2*, *clb19*, *ys1*, and *otp70* (Pfalz et al., 2006; Chateigner-Boutin et al., 2008; Zhou et al., 2009; Chateigner-Boutin et al., 2011). We also found there was a lack of correlation between gene expression and protein accumulation for some class I (such as psbA) and class III (rpoB) products. This discrepancy between transcript and protein levels may be due to feedback regulation or post-transcriptional modification.

RNA splicing is the process of removing introns between neighboring exons during translation (Lorkoviæ et al., 2000). Many RNA binding proteins have essential roles in RNA splicing of introns in plant plastids, including maturase proteins, CRM (Chloroplast RNA splicing and ribosome maturation) proteins, PPR proteins, and other nuclear-encoded factors (de Longevialle et al., 2010). To date, five PPR proteins have been reported to be involved in RNA splicing of group II introns in chloroplasts. Among them, the maize PPR4 protein acts as an *rps12 trans*-splicing factor (Schmitz-Linneweber et al., 2006), and PPR5, a maize P-class PPR protein, stabilizes *trnG*-UCC tRNA precursors in chloroplasts by binding and protecting the endonuclease-sensitive site; its mutation indirectly influences splicing of *trnG*-UCC RNAs (Beick et al., 2008). The Arabidopsis PPR protein OTP51 functions as a plastid *ycf3-*2 intron *cis*-splicing factor (de Longevialle et al., 2008). The Arabidopsis E-class PPR protein OTP70 has been implicated in splicing of the plastid transcript *rpoC1* (Chateigner-Boutin et al., 2011). The maize short PPR protein THA8 is associated with splicing of specific *ycf3-2* and *trnA* group II introns in chloroplasts (Khrouchtchova et al., 2012). In this study, the *wsl4* mutant caused defects in the splicing of *atpF, ndhA, rpl2*, and *rps12* (**Figure 9**), implying that WSL4 probably involves chloroplast RNA intron splicing during early leaf development in rice. To confirm this, further studies will be needed in future. Subcellular localization showed that WSL4 protein is a chloroplast nucleoid-localized protein (**Figure 4**), suggesting that it may participate in plastid RNA metabolism.

Plastidial maturase MatK is involved in RNA splicing by interacting with seven group II introns including *trnV*, *trnI*, *trnA*, *trnK*, *atpF*, *rpl2*, and *rps12* intron 2 (Zoschke et al., 2010). It is possible that the splicing defects of *atpF*, *rpl2*, and *rps12* transcripts (**Figure 9**) are due to lack of plastidial maturase MatK in the *wsl4* mutant. A similar phenomenon was observed in the Arabidopsis *emb2654* mutant (Aryamanesh et al., 2017). Given that *ndhA* is not a target of MatK, it is likely that WSL4 functions in the splicing of *ndhA* directly. Similar RNA splicing events of *ndhA* were observed in many other Chl deficient mutants, such as *emb2654*, *ppr53*, *otp70*, *clb19*, and *sot1* (Chateigner-Boutin et al., 2008; Chateigner-Boutin et al., 2011; Wu et al., 2016; Zoschke et al., 2016; Aryamanesh et al., 2017). Therefore, it is also possible that the splicing defect of *ndhA* is a secondary effect of disrupted chloroplast development or reduced PEP activity in the *wsl4* mutant.

RNA editing is a post-transcriptional process that alters RNA sequences by converting specific target cytidines to uridine in both plastid and mitochondrial transcripts (Takenaka et al., 2013). Our results showed that the editing efficiency of *rpoB* at C545 and C560 in WT is relatively low (15 and 20%, respectively), but almost complete in the *wsl4* mutant (75 and 90%, respectively) (**Figure 7** and **Supplementary Figure S6**). This inverse relationship between *rpoB* editing and the presence of *WSL4* makes it extremely unlikely that WSL4 has a direct role in editing of *rpoB*. This phenotypic defect is most reminiscent of the previously reported *iojap* mutant in maize (that lacks chloroplast ribosomes) in which the editing sites in *rpoB* are highly edited (Halter et al., 2004). A possible explanation is that the lack of plastid translation may be indirectly responsible for efficient *rpoB* editing. In the Arabidopsis PEP-deficient mutant *otp70* editing efficiency of unspliced *rpoC1* transcripts is almost complete, but is relatively low in WT (Chateigner-Boutin et al., 2011). A reasonable explanation is that the editing of *rpoC1* in *otp70* mutant is influenced by the changes in splicing. Therefore, altered editing is most likely a secondary effect of defective splicing of chloroplast group II introns in the *wsl4* mutant. Further studies on searching for interacting partners of WSL4- will help to uncover the regulatory mechanism of chloroplast development during the early leaf development.

## Author Contributions

JW and YiW conceived the research. YiW, KZ, YR, YX, HZ, LZ, ZF, LW, and WM performed the experiments. JW provided the mutant material. YR, JW, XG, XZ, CL, YuW, and ZC provided the technical assistance. YiW, YR, and LL analyzed the data and wrote the manuscript. All authors declared no conflicting interest on the contents of the manuscript.

## Conflict of Interest Statement

The authors declare that the research was conducted in the absence of any commercial or financial relationships that could be construed as a potential conflict of interest.

## Footnotes

1 http://www.gramene.org/

2 www.gramene.org/

3 http://www.ncbi.nlm.nih.gov/

4 http://www.cbs.dtu.dk/services/ChloroP/

5 http://www.cbs.dtu.dk/services/TargetP/

6 http://www.megasoftware.net/

7 http://www.genomics.cn/index

8 http://www.cbs.dtu.dk/services/ChloroP/

9 http://www.cbs.dtu.dk/services/TargetP/

10 http://rice.plantbiology.msu.edu/cgi-bin/gbrowse/rice/

11 http://ricexpro.dna.affrc.go.jp/GGEP/

12 http://www.shigen.nig.ac.jp/rice/oryzabase/top/top.jsp

## Supplementary Material

**FIGURE S1 |** Amino acid sequence alignment of the WSL4 and wsl4 proteins. Premature translation in the *wsl4* mutant generated a truncated protein lacking 111 amino acids residues at the C-terminal, compared to WT WSL4 protein.

**FIGURE S2 |** Phylogenetic tree of WSL4 and its homologs in plants. Numbers near the major branches are bootstrap values. GenBank accession numbers for these proteins are: XP_010235566.1 (*Brachypodium distachyon*), BAK03057.1 (*Hordeum vulgare*), XP_004952727.1 (*Setaria italica*), NP_001141010.1 (*Zea mays*), KXG30467.1 (*Sorghum bicolor*), KQL30111.1 (*Setaria italica*), EMT15761.1 (*Aegilops tauschii*), XP_010917143.1 (*Elaeis guineensis*), XP_009402131.1 (*Musa acuminata* subsp. *malaccensis*), XP_015886049.1 (*Ziziphus jujuba*), XP_009800134.1 (*Nicotiana sylvestris*), XP_017981749.1 (*Theobroma cacao*), XP_008444958.1 (*Cucumis melo*), XP_010104578.1 (*Morus notabilis*), XP_002452305.1 (*Sorghum bicolor*), XP_011071341.1 (*Sesamum indicum*), XP_012840102.1 (*Erythranthe guttata*), XP_010671063.1 (*Beta vulgaris* subsp. *vulgaris*), XP_015935032.1 (*Arachis duranensis*), XP_017611896.1 (*Gossypium arboreum*), XP_016163747.1 (*Arachis ipaensis*), XP_009366374.1 (*Pyrus x bretschneideri*), XP_011649703.1 (*Cucumis sativus*), XP_010254618.1 (*Nelumbo nucifera*), XP_016576050.1 (*Capsicum annuum*), XP_012450936.1 (*Gossypium raimondii*), XP_016576051.1 (*Capsicum annuum*), XP_006433766.1 (*Citrus clementina*), XP_010321813.1 (*Solanum lycopersicum*), XP_016163746.1 (*Arachis ipaensis*), XP_006472405.1 (*Citrus sinensis*), XP_017189272.1 (*Malus domestica*), XP_015078222.1 (*Solanum pennellii*), XP_006364562.1 (*Solanum tuberosum*), XP_017178849.1 (*Malus domestica*), XP_002285611.1 (*Vitis vinifera*), XP_016464120.1 (*Nicotiana tabacum*), XP_008219211.2 (*Prunus mume*), XP_010544054.1 (*Tarenaya hassleriana*), XP_011016979.1 (*Populus euphratica*), XP_009595574.1 (*Nicotiana tomentosiformis*), XP_015579793.1 (*Ricinus communis*), XP_013696710.1 (*Brassica napus*), XP_014502416.1 (*Vigna radiata var. radiata*), XP_002889775.1 (*Arabidopsis lyrata*), XP_010061566.1 (*Eucalyptus grandis*), NP_172461.1 (*Arabidopsis thaliana*), XP_017422523.1 (*Vigna angularis*), XP_013640951.1 (*Brassica napus*), XP_009148305.1 (*Brassica rapa*), XP_010490447.1 (*Camelina sativa*), XP_011627875.1 (*Amborella trichopoda*), XP_004288876.1 (*Fragaria vesca*), XP_017249274.1 (*Daucus carota*), XP_013585784.1 (*Brassica oleracea*), XP_003523769.1 (*Glycine max*), XP_017244771.1 (*Daucus carota*), XP_003527866.1 (*Glycine max*), XP_012068070.1 (*Jatropha curcas*), XP_004501057.1 (*Cicer arietinum*), XP_006306156.1 (*Capsella rubella*), KVH98829.1 (*Cynara cardunculus* var), KYP46243.1 (*Cajanus cajan*), KHN08231.1 (*Glycine soja*), KMZ57347.1 (*Zostera marina*), ERN18052.1 (*Amborella trichopoda*).

**FIGURE S3 |** Amino acid sequence alignment of WSL4 and its close homologs. Sequences are from *Oryza sativa* WSL4, *Brachypodium distachyon* XP_010235566.1, *Hordeum vulgare* BAK03057.1, *Sorghum bicolo*r XP_002452305.1, *Sorghum bicolor* KXG30467.1, Zea mays NP_001141010.1, *Setaria italica* XP_004952727.1, *Setaria italica* KQL30111.1, *Aegilops tauschii* EMT15761.1, and *Arabidopsis thaliana* NP_172461.1.

**FIGURE S4 |** Quantitative RT-PCR analyses of genes related to chlorophyll biosynthesis in WT and the *wsl4* mutant. RNA was extracted from L3-3 of WT and *wsl4* mutant plants grown in a growth chamber under L30/D25. L3-3 indicates the third leaf at the three-leaf stage. Values are means ± SD of three replicates. (Student’s *t*-test: *^∗^P* < 0.05, *^∗∗^P* < 0.01).

**FIGURE S5 |** RNA editing analyses of chloroplast genes in WT and *wsl4* mutant. Arrowheads indicate editing sites and their positions in the corresponding chloroplast cDNA strand. RNA was extracted from L3-3 of WT and *wsl4* mutant (white striped sector) plants grown in a growth chamber at L30/D25. L3-3 indicates the third leaf at the three-leaf stage.

**FIGURE S6 |** Low temperature exacerbated splicing defects of *atpF*, *ndhA*, *rpl2*, and *rps12* transcripts in *wsl4* mutant. qRT-PCR analyses of *atpF*, *ndhA*, *rpl2*, and *rps12* transcripts in L3-3 from WT and *wsl4* mutant (white striped sectors) seedlings grown in a growth chamber with 12 h photoperiod at C30, L30/D25, and C20. L3-3 indicates the third leaf at the three-leaf stage. Histograms show log2 ratios of spliced to unspliced RNA in *wsl4* compared to WT. Values are means ± SD of three replicates. (Student’s *t*-test: *^∗^P* < 0.05, ^∗∗^*P* < 0.01).

## Abbreviations

Chl: chlorophyll
GFP: green fluorescent protein
PPR: pentatricopeptide repeat
RNAi: RNA interference
RT–PCR: reverse transcription–PCR
TEM: transmission electron microscopy
WT: wild type
