# Supplementary figures and images for: WHITE STRIPE LEAF4 Encodes a Novel P-Type PPR Protein Required for Chloroplast Biogenesis during Early Leaf Development
Source: Front Plant Sci. 2017 Jun 26;8:1116. doi: 10.3389/fpls.2017.01116 (PMC5483476; doi:10.3389/fpls.2017.01116)

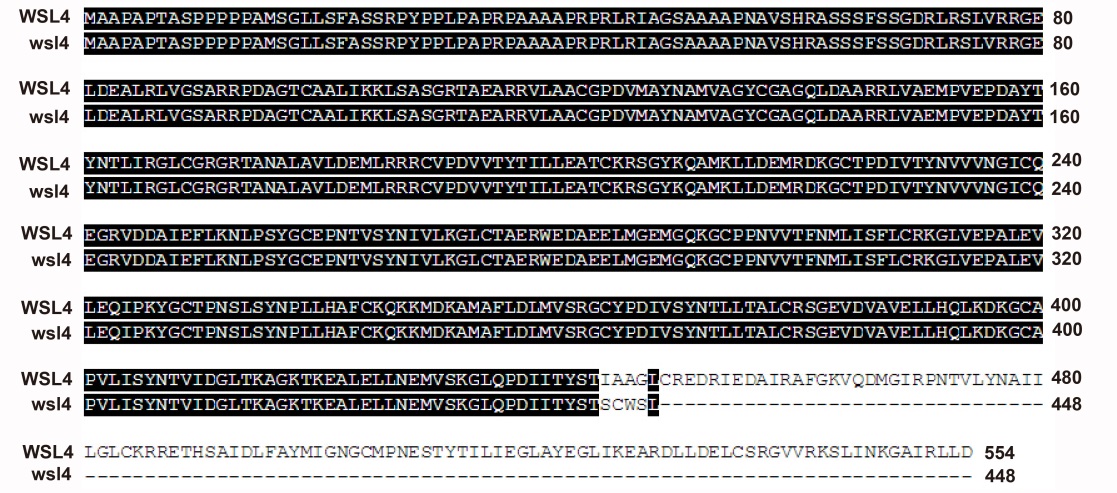

Supplement: Supplementary file 1 [file Image_1.JPEG]

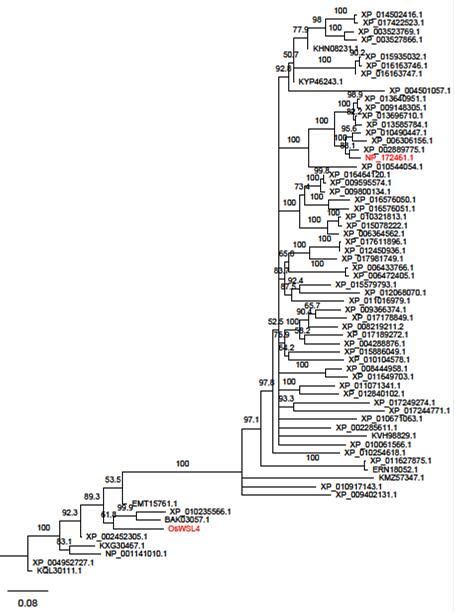

Supplement: Supplementary file 2 [file Image_2.JPEG]

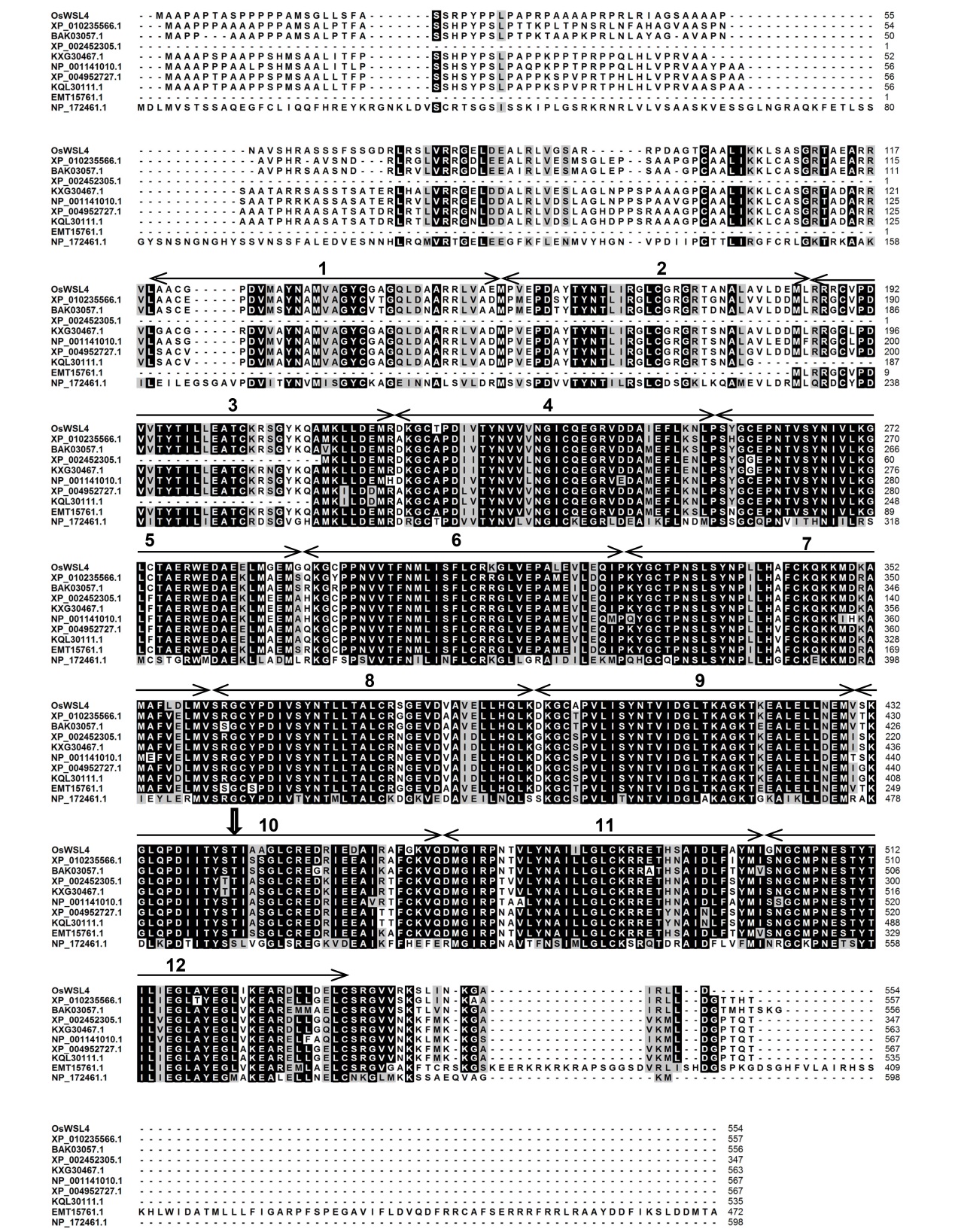

Supplement: Supplementary file 3 [file Image_3.JPEG]

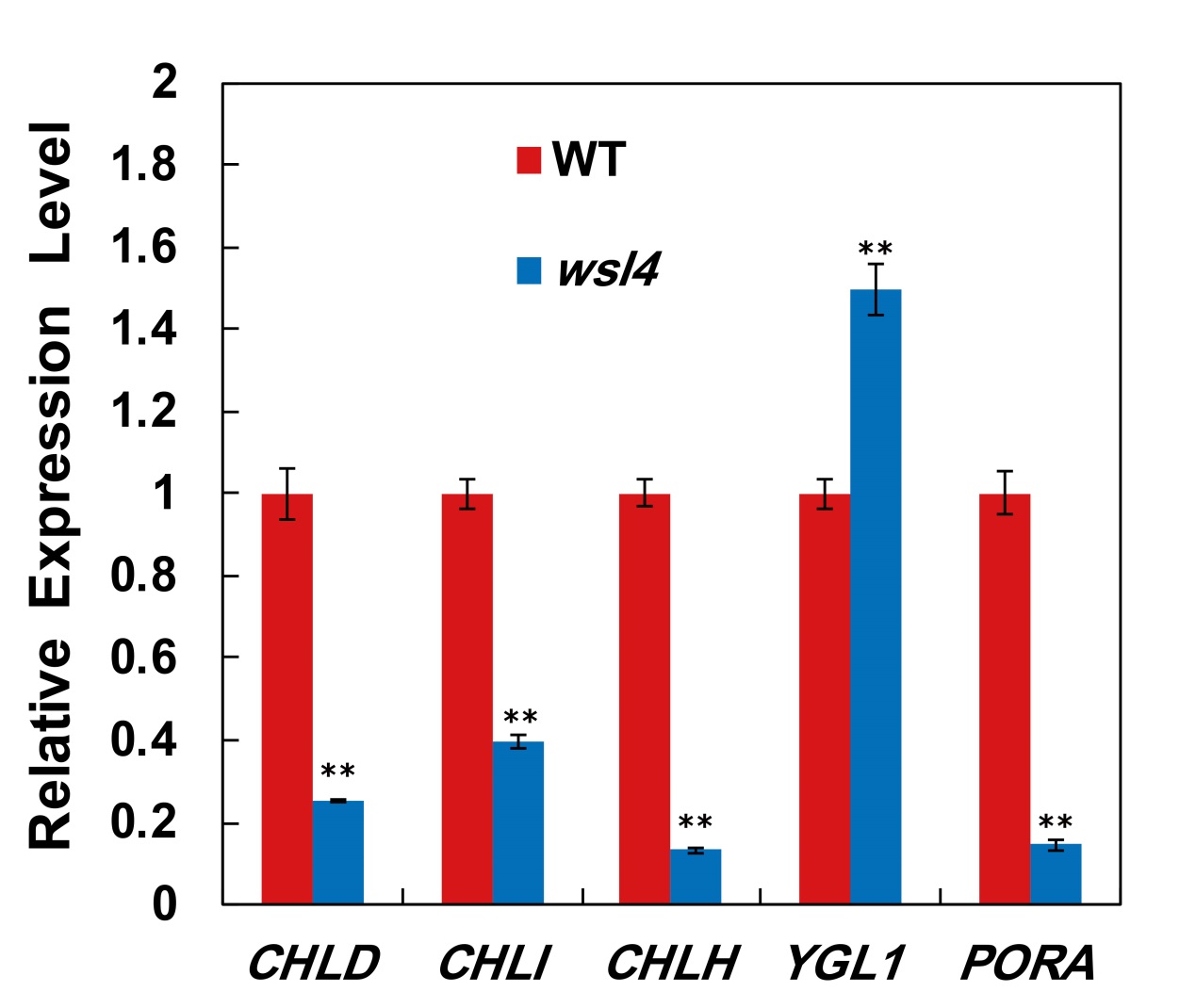

Supplement: Supplementary file 4 [file Image_4.JPEG]

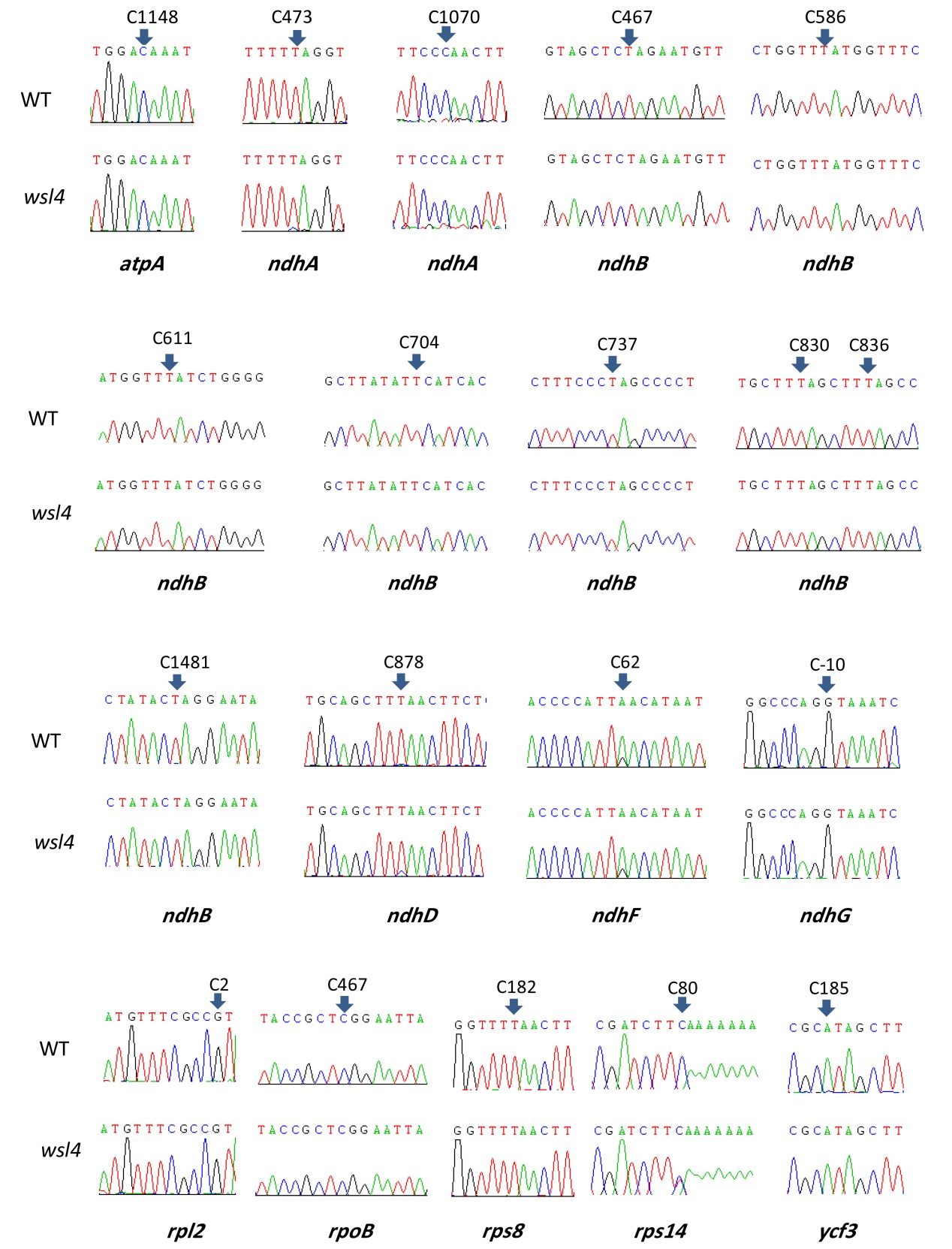

Supplement: Supplementary file 5 [file Image_5.JPEG]

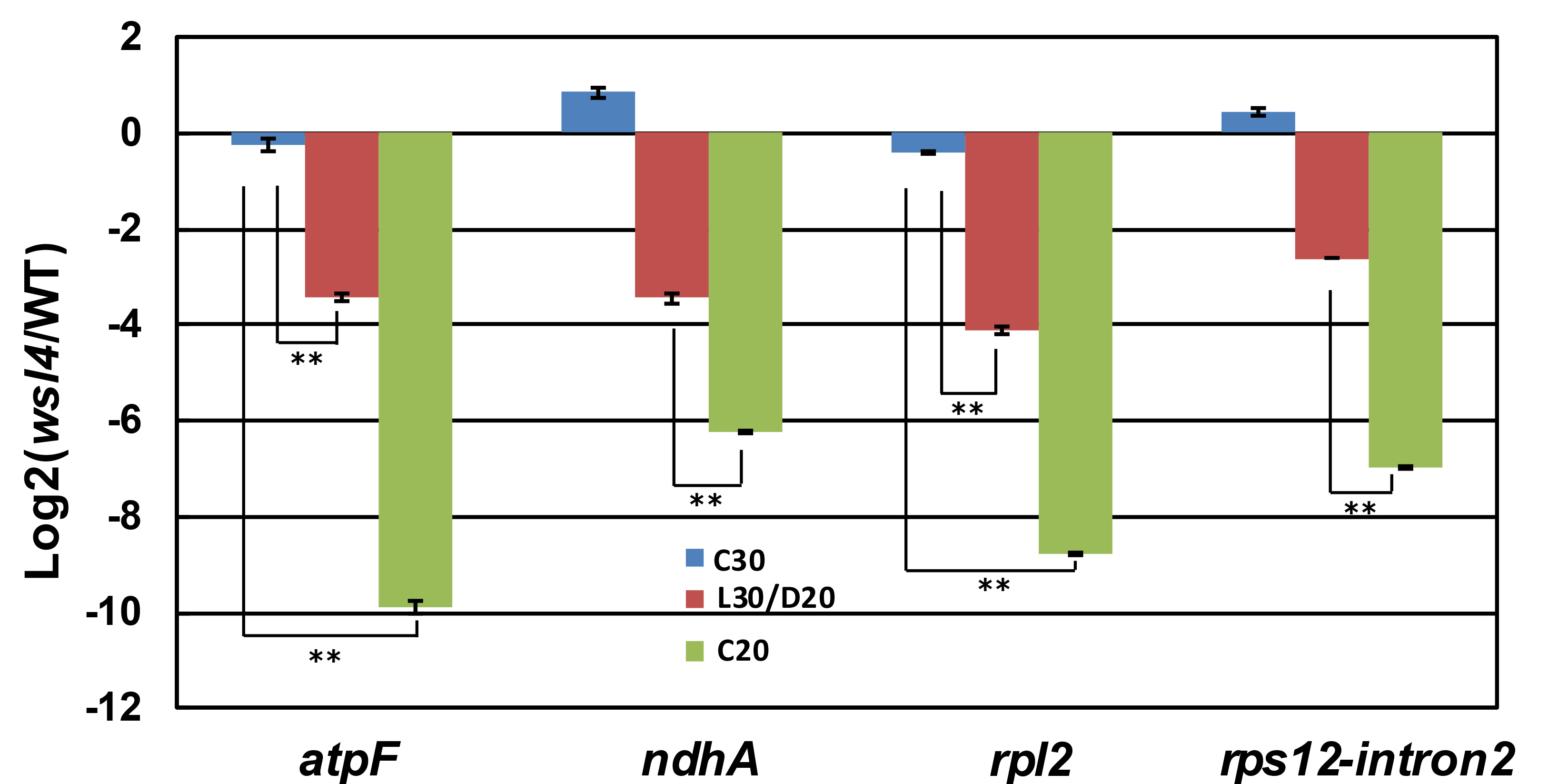

Supplement: Supplementary file 6 [file Image_6.JPEG]
