# Supplementary material for: WHITE STRIPE LEAF4 Encodes a Novel P-Type PPR Protein Required for Chloroplast Biogenesis during Early Leaf Development
Source: Front Plant Sci. 2017 Jun 26;8:1116. doi: 10.3389/fpls.2017.01116 (PMC5483476; doi:10.3389/fpls.2017.01116)
Supplement: Supplementary file 7 [file Table_1.DOCX]

***Supplementary Material***

***WHITE STRIPE LEAF4* encodes a novel P-type PPR protein required for chloroplast biogenesis during early leaf development**

*Ying Wang^a,1^, Yulong Ren^a,1^, Kunneng Zhou^a,1^, Linglong Liu^b,1^, Jiulin Wang^a^, Yang Xu^b^, Huan Zhang^b^, Long Zhang^a^, Zhiming Feng^b^, Liwei Wang^b^, Weiwei Ma^a^, Yunlong Wang^b^, Xiuping Guo^a^, Xin Zhang^a^, Cailin Lei^a^, Zhijun Cheng^a^ and Jianmin Wan^a,b,^**

*^a^ National Key Facility for Crop Gene Resources and Genetic Improvement, Institute of Crop Science, Chinese Academy of Agricultural Sciences, Beijing 100081, P.R. China*

*^b^* *National Key Laboratory for Crop Genetics and Germplasm Enhancement, Nanjing Agricultural University, Nanjing 210095,* *P.R. China*

^1^ These authors contributed equally to this manuscript

* To whom correspondence should be addressed.

Corresponding author: Jianmin Wan

Address: Institute of Crop Sciences Chinese Academy of Agriculture Sciences, 12#

Zhongguancun South Street, Haidian District, Beijing, 100081, China, P. R.

Phone: 0086-10-82105837

E-mail: [wanjianmin@caas.cn](mailto:wanjianmin@caas.cn)

**Supplementary Table S1** List of primer pairs used in this study

| **Usage** | **Primer name** | **Sequence (5'-3')** |
| --- | --- | --- |
| **Gene mapping** | L-3-F | GAGATATATCATCCCGAGGAAAA |
|  | L-3-R | GGCAGGCACAGATAGTTTCG |
|  | L-9-F | CTCCGGTAAGAAGACAGATCG |
|  | L-9-R | CAATTTCAGCCAGTGGGATT |
|  | L-19-F | CTTCCTTTTCTGCCCATGC |
|  | L-19-R | TCACCATCTGTGCTGTCACC |
|  | L-21-F | CGCGTGAGTTTTCTATTCCA |
|  | L-21-R | GATTATTATCGCGGGGGTTT |
|  | L-26-F | GCCATCTACTGTGCGTGTCA |
|  | L-26-R | CCTGCCGACCTTTATAGCAA |
|  | L-29-F | CGAACGAAGCAAAGATGTGA |
|  | L-29-R | TCAGGGGGATATGAAGCAAC |
|  | L-35-F | GTCGCATTCTCGCAACGG |
|  | L-35-R | TTTGGCTATGCTTCATGGATTT |
|  | L-37-F | CCCGTAAAGTGTTTCAGCC |
|  | L-37-R | ATGGCACTTGGAAGGTCTG |
| **Plasmid construction** | PPR-G-F | ATGGCGTATCCTCCTATCGTTGC |
|  | PPR-G-R | GCCGACTTCCGACCGAACAT |
|  | *WSL4*-RNAi-SacI-InF | CTAGGTACCAGGCCTGAGCTCGTTGAATTGCTTCATCAA |
|  | *WSL4*-RNAi-SacI-InR | GACGTAGGGGCGATAGAGCTTAAGCGAAAAGATCAATG |
|  | *WSL4*-RNAi-BamHI-InF | TCTTAGAATTCCCGGGGATCCGTTGAATTGCTTCATCAA |
|  | *WSL4*-RNAi-BamHI-InR | CGTTACGTAGTCGACGGATCCTAAGCGAAAAGATCAAT |
|  | WSL4-GFP-InXbaI | CGGAGCTAGCTCTAGAATGGCCGCGCCCGCGCCCA |
|  | WSL4-GFP-InBamHI | TGCTCACCATGGATCCATCAAGCAGTCTAATGGCT |
|  | wsl4-GFP-InXbaI | CGGAGCTAGCTCTAGAATGGCCGCGCCCGCGCCCA |
|  | wsl4-GFP-InBamHI | TGCTCACCATGGATCCATTCTATCTTCTCTACAA |
| **Editing analysis** | *atpA*-F | CCCAGGGGATGTTTTTTATT |
|  | *atpA*-R | TGAAAAAAGCGTCCATTGTC |
|  | *ndhA*-F | ATGATAATAGACAGGGTACAGG |
|  | *ndhA*-R | TTATAGTGAAACAAGTTGGGAAG |
|  | *ndhB*-F | ATGATCTGGCATGTACAGAATG |
|  | *ndhB*-R | CTAAAAGAGGGTATCCTGAGCA |
|  | *ndhD*-F | ATTTTGGCTTCCTTATTGC |
|  | *ndhD*-F | GCCTCTACCCTGTCAACG |
|  | *ndhF*-F | ATATGCATGGGTAATCCCTC |
|  | *ndhF*-R | AGTGGCTCCTAAGAAAAGTG |
|  | *ndhG*-F | ATGGATTTACCTGGGCCAAT |
|  | *ndhG*-R | TTATTGCCGAGCCATAGTAA |
|  | *rpl2*-F | ACGGCGAAACATTTATACAA |
|  | *rpl2*-R | TTACTTACGGCGACGAAGAATA |
|  | *ropB*-F | ACTAAGCGTGCTATTCTCAA |
|  | *ropB*-R | TTTATGGTCTAATTCCGAGC |
|  | *rps8*-F | ATGGGCAAGGACACTATTG |
|  | *rps8*-R | AACATAAGACTTCTCCCCCA |
|  | *rps14*-F | ATGGCAAAAAAAAGTTTGATTC |
|  | *rps14*-R | TTACCAACTGGATCTTGTTGCA |
|  | *ycf3*-F | ATGCCTAGATCCCGTATAAATG |
|  | *ycf3*-R | TTATTCAAATTCAAAGCGCTTC |
| **Splicing analysis** | *atpF*-F | ATGAAAAATGTAACCCATTCTT |
|  | *atpF*-R | AAGAATGGGTTACATTTTTCAT |
|  | *ndhA* | ATGATAATAGACAGGGTACAGG |
|  | *ndhA* | TTATAGTGAAACAAGTTGGGAAG |
|  | *ndhB* | ATGATCTGGCATGTACAGAATG |
|  | *ndhB* | CTAAAAGAGGGTATCCTGAGCA ' |
|  | *petB* | TTCTCATATACGGTTCTCGG |
|  | *petB* | TAAAGGGCCCGAAATACCTT |
|  | *petD* | ATGGGAGTAACAAAGAAACC |
|  | *petD* | TGTTGCTCCAATACCTAACC |
|  | *rpl2* | ACGGCGAAACATTTATACAA |
|  | *rpl2* | TTACTTACGGCGACGAAGAATA |
|  | *rpl16* | ATGCTTAGTCCCAAAAGAAC |
|  | *rpl16* | AACCGAAGAAATTGACTTCG |
|  | *rps12- intron 1* | ACTATCAACCCCAAAAAACC |
|  | *rps12- intron 1* | TTTGGCTTTTTGACCCCAT |
|  | *rps16* | AAAACGATGTGGTAGAAAGC |
|  | *rps16* | AGAATTCCGCCTTCCTTAAA |
|  | *trnA* | GGGGATATAGCTCAGTTGGT |
|  | *trnA* | TGGAGATAAGCGGACTCGAA |
|  | *trnG* | TCGTTAGCTTGGAAGGCTAG |
|  | *trnG* | GCGGGTATAGTTTAGTGGTA |
|  | *trnI* | TGGGCCATCCTGGACTTGA |
|  | *trnI* | AGCTCAGTGGTAGAGCGCG |
|  | *trnK* | GGTTGCCCGGGACTCGAA |
|  | *trnK* | GGGTTGCTAACTCAATGGTAGAG |
|  | *trnL* | GGATATGGCGAAATCGGTA |
|  | *trnL* | TGGGGATAGAGGGACTTGA |
|  | *trnV* | TAGGGCTATACGGATTCGAA |
|  | *trnV* | AGGGCTATAGCTCAGTTCGG |
|  | *ycf3* | ATGCCTAGATCCCGTATAAATG |
|  | *ycf3* | TTATTCAAATTCAAAGCGCTTC |
|  | *23S* | TTCAAAAGAGGAAAGGCTTG |
|  | *23S* | AGAGAGCACTCATCTTGGGG |
| **RT-PCR analysis** | *rpl2-U*-F | TTGGAGATACTATTGTTTCTGGTACA |
|  | *rpl2-U*-R | CGTAAACCTAATTGGTTACTTCCA |
|  | *rpl2-S*-F | ATCAATGGGAAATGCCCTAC |
|  | *rpl2-S*-R | TGCTGCTCTAGCTAATTGCC |
|  | *rps12- intron 1-U*-F | GAGGAGCCCTAGATGCTGTC |
|  | *rps12- intron 1-U*-R | TTTCTAGCGATTCACATGGC |
|  | *rps12- intron 1-S*-F | AAGGATTTACCCGGTGTGAG |
|  | *rps12- intron 1-S*-R | CCCATATTTAGAACGCCCTT |
|  | *ndhA-U*-F | GAAACTAAGGTTTCATGTACGGTTT |
|  | *ndhA-U*-R | TGAACTGTTGGATAATCATAGTCG |
|  | *ndhA -S*-F | TTGGTCTTCTTATGGCAGGA |
|  | *ndhA -S*-R | TGAACTGTTGGATAGTAGAGATATTGC |
|  | *atpF-U*-F | CTGTAGTGGTTGGTGTATTGATTTATT |
|  | *atpF-U*-R | TTCTAAAGTGCAGCCGGATA |
|  | *atpF –S*-F | ACTGTAGTGGTTGGTGTATTGATTT |
|  | *atpF –S*-R | TTTCGAGCTGCTCAATGGT |
|  | *WSL4*-*RT*-F | GCCCTCCAAATGTGGTAACT |
|  | *WSL4-RT*-R | GCCATCGCTTTATCCATCTT |
|  | *CHLD*-F | GGAAAGAGAGGGCATTAG |
|  | *CHLD*-R | CAATACGATCAAGTAAGTGTT |
|  | *CHLI*-F | AGTAACCTTGGTGCTGTG |
|  | *CHLI*-R | AATCCATCAACATTCAACTCTG |
|  | *CHLH*-F | CTATACATTCGCCACACT |
|  | *CHLH*-R | TATCACACAACTCCCAAG |
|  | *YGL1*-F | TGGACAGTTGAAGATGTT |
|  | *YGL1-R* | GAATAGGACGGTAAGGTT |
|  | *PORA-F* | ATCACCAAGGGCTACGTCTC |
|  | *PORA-R* | GAGTTGTTGTTCCAGCTCCA |
|  | *cab1R*-F | AGACGTTCGCCAAGAACC |
|  | *cab1R*-R | GAGGAGCTCCGGGAAGAC |
|  | *cab2R*-F | GTTCTCCATGTTCGGCTTCT |
|  | *cab2R*-*R* | GACGAAGTTGGTGGCGTAG |
|  | *psaA*-F | GAGATACCACTTCCTCAT |
|  | *psaA*-R | ACTAAGAAATTCTGCGTATT |
|  | *psbA*-F | AAGTTTCTCTGATGGTATG |
|  | *psbA*-R | ATAGCACTGAATAGGGAA |
|  | *psbC*-F | TACAACCTTGGCAAGAACGA |
|  | *psbC*-R | TACGCCACCCACAGAATTTA |
|  | *rbcS*-F | TCATCAGCTTCATCGCCTAC |
|  | *rbcS*-R | ACTGGGAACACACGAAACAA |
|  | *psaB*-F | TTGGTATTGCTACCGCACAT |
|  | *psaB*-F | CCGGACGTCCATAGAAAGAT |
|  | *psbB-F* | TCATATTGCTGCGGGTACAT |
|  | *psbB-R* | AGTTGCTGACCCATACCACA |
|  | *rbcL-F* | GTTGAAAGGGATAAGTTGA |
|  | *rbcL*-R | AATGGTTGTGAGTTTACG |
|  | *Ubq*-F | GCTCCGTGGCGGTATCAT |
|  | *Ubq*-R | CGGCAGTTGACAGCCCTAG |
| **Northern blot analysis** | *rpl2-T*-F | TGCTGTAGCGAAACTGATTG |
|  | *rpl2-T*-R | AATTAACCCTCACTAAAGGGACGGCGACGAAGAATAAA |
|  | *rps12* -*intron 1- T*-F | AAAACCCAACTCTGCCTTACG |
|  | *rps12* -*intron 1- T*-R | AATTAACCCTCACTAAAGGGTAGAACGCCCTTGTTGACG |
|  | *ndhA- T-*F | CAGAAGCGGAGGAAGAAT |
|  | *ndhA- T-*R | AATTAACCCTCACTAAAGGGTCTCATCCTGGGTAAAGTCC |
|  | *atpF- T*-F | GAATCAGGTCCGACAACG |
|  | *atpF- T*-R | AATTAACCCTCACTAAAGGGGGTTGCCATTAGTGTTTCTT |
